# Metabolic effects of nuclear receptor activation in vivo after 28-day oral exposure to three endocrine-disrupting chemicals

**DOI:** 10.1007/s00204-023-03658-2

**Published:** 2024-01-05

**Authors:** Brecht Attema, Outi Kummu, Sini Pitkänen, Jonna Weisell, Taina Vuorio, Erika Pennanen, Maria Vorimo, Jaana Rysä, Sander Kersten, Anna-Liisa Levonen, Jukka Hakkola

**Affiliations:** 1 Nutrition, Metabolism and Genomics Group, Division of Human Nutrition and Health, Wageningen University, Wageningen, The Netherlands; 2 Research Unit of Biomedicine and Internal Medicine, Biocenter Oulu, Medical Research Center Oulu, University of Oulu and Oulu University Hospital, Oulu, Finland; 3 A.I. Virtanen-Institute for Molecular Sciences, University of Eastern Finland, Kuopio, Finland; 4 Finnish Institute of Occupational Health, Kuopio, Finland; 5 School of Pharmacy, University of Eastern Finland, Kuopio, Finland

**Keywords:** Endocrine-disrupting chemicals (EDCs), Nuclear receptors, Metabolic disruption, Metabolism-disrupting chemicals, Glucose metabolism, Lipid metabolism, Hepatic steatosis

## Abstract

Environmental exposure to endocrine-disrupting chemicals (EDCs) can lead to metabolic disruption, resulting in metabolic complications including adiposity, dyslipidemia, hepatic lipid accumulation, and glucose intolerance. Hepatic nuclear receptor activation is one of the mechanisms mediating metabolic effects of EDCs. Here, we investigated the potential to use a repeated dose 28-day oral toxicity test for identification of EDCs with metabolic endpoints. Bisphenol A (BPA), pregnenolone-16α-carbonitrile (PCN), and perfluorooctanoic acid (PFOA) were used as reference compounds. Male and female wild-type C57BL/6 mice were orally exposed to 5, 50, and 500 μg/kg of BPA, 1000, 10 000, and 100 000 µg/kg of PCN and 50 and 300 μg/kg of PFOA for 28 days next to normal chow diet. Primary endpoints were glucose tolerance, hepatic lipid accumulation, and plasma lipids. After 28-day exposure, no changes in body weight and glucose tolerance were observed in BPA-, PCN-, or PFOA-treated males or females. PCN and PFOA at the highest dose in both sexes and BPA at the middle and high dose in males increased relative liver weight. PFOA reduced plasma triglycerides in males and females, and increased hepatic triglyceride content in males. PCN and PFOA induced hepatic expression of typical pregnane X receptor (PXR) and peroxisome proliferator-activated receptor (PPAR)α target genes, respectively. Exposure to BPA resulted in limited gene expression changes. In conclusion, the observed changes on metabolic health parameters were modest, suggesting that a standard repeated dose 28-day oral toxicity test is not a sensitive method for the detection of the metabolic effect of EDCs.

## Introduction

Endocrine disruptors (EDCs) are chemicals that interfere with hormone actions (Zoeller et al. 2012). EDCs can be found in many products used in everyday life, such as plasticware, detergents, cosmetics, textiles, and also in food and pharmaceuticals. The main routes of EDC exposure in humans are via ingestion, inhalation, and transdermal uptake (Gore et al. 2015).

Exposure to certain EDCs may predispose to a variety of metabolic disturbances. In this context, the term obesogen has been coined recently, referring to EDCs that stimulate adipogenesis and lipid accumulation and may promote obesity (Heindel et al. 2015; Darbre 2017). EDCs have also been linked to the development of type 2 diabetes and related metabolic disturbances, i.e., metabolic syndrome and non-alcoholic fatty liver disease (NAFLD) (Heindel et al. 2017; Haverinen et al. 2021). The chemicals that have been associated with detrimental metabolic effects are also referred to as metabolism-disrupting chemicals (MDCs). One of the main mechanisms for metabolic disruption is the modulation of nuclear receptor function. Nuclear receptors are transcription factors that regulate the expression of numerous genes involved in a variety of physiological functions, including energy and lipid metabolism.

Today, there are no established, standard toxicity testing approaches to evaluate MDCs. Here, we investigated the possibility of utilizing a repeated dose 28-day oral toxicity study (OECD 2008) for the identification and characterization of MDCs. To test this approach, we selected three study compounds with multiple reports in the literature suggesting metabolism-disrupting characteristics and different nuclear receptor preferences.

Bisphenol A (BPA) is an industrial chemical ubiquitously present in our environment (Vandenberg et al. 2010). It is used as a plasticizing agent in polycarbonate plastics and epoxy resins and is a common raw material for consumer products such as food packages, containers, and toys. BPA is widely recognized as an environmental estrogen, having an affinity for both estrogen receptor isoforms, ERα (NR3A1) and ERβ (NR3A2) (Liu et al. 2019). In addition to the ERs, BPA has been suggested to modulate functions of other nuclear receptors involved in metabolic regulation, including the peroxisome proliferator-activated receptors (PPARs), estrogen-related receptor γ (ERRγ), liver X receptors (LXRs), and thyroid hormone receptor (THR) (Moriyama et al. 2002; Tohmé et al. 2014; Ariemma et al. 2016; Ji et al. 2020). Studies in cell and animal models have suggested that BPA can disrupt metabolism and promote obesity (Le Corre et al. 2015; Legeay and Faure 2017). For example, oral low-dose exposure to BPA upregulated genes related to lipid synthesis and promoted liver triglyceride accumulation in a 28-day oral toxicity test in CD-1 male mice (Marmugi et al. 2012). Moreover, in different types of rodent studies involving single intraperitoneal, perinatal, and long-term oral dosing, BPA was reported to promote hyperinsulinemia, reduce glucose and insulin tolerance, and disrupt pancreatic beta-cell function (Alonso-Magdalena et al. 2006; Liu et al. 2013; Moon et al. 2015).

Another group of EDCs with metabolism-disrupting properties is the poly- and perfluoroalkyl substances (PFAS) (Intrasuksri et al. 1998; Takacs and Abbott 2007; Fragki et al. 2021). PFAS, which include perfluorooctanoic acid (PFOA), are known to activate the nuclear receptor peroxisome proliferator-activated receptor α (PPARα, NR1C1). PPARα is the master transcriptional regulator of lipid metabolism in the liver (Kersten 2014). PPARα is known to be activated by endogenous ligands such as fatty acids and eicosanoids, as well as by numerous synthetic agonists such as fibrates, phthalates, and PFAS (Krey et al. 1997; Murakami et al. 1999). Indeed, multiple studies have reported on the activation of both mouse and human PPARα by PFOA (Takacs and Abbott 2007; Bjork et al. 2011). Recently, we demonstrated that exposing mice to PFOA enhances hepatic lipid accumulation and reduces plasma triglycerides and cholesterol in mice fed a high-fat diet (Attema et al. 2022).

Finally, we wanted to include the environmental xenosensor pregnane X receptor (PXR, NR1I2) in this analysis because of its suspected important role in metabolic disruption. PXR is involved in the regulation of glucose and lipid metabolism (Hakkola et al. 2016; Zhou 2016). PXR activation has been found to impair glucose tolerance both in rodents and humans (Hassani-Nezhad-Gashti et al. 2018; Rysä et al. 2013), induce cholesterol synthesis in obese mice (Karpale et al. 2021), and suppress hepatic fatty acid oxidation and ketogenesis, while overexpression of PXR in the liver markedly enhanced hepatic lipid accumulation (Nakamura et al. 2007; Zhou et al. 2006). PXR has a large and flexible binding pocket that can accommodate a variety of different ligands, including environmental chemicals, herbal remedies, and medicinal drugs. However, the PXR ligand preference is highly species-specific. Therefore, compound-specific results cannot be directly transferred from experimental animals to humans. In this study, we used a rodent-specific PXR-ligand pregnenolone-16α-carbonitrile (PCN) as a model compound.

With these three compounds, BPA, PCN, and PFOA, which primarily target ER, PXR, PPARα, respectively, we investigated metabolic endpoints such as weight gain, glucose tolerance, liver fat accumulation, and plasma lipids after 28 days of oral exposure in mice and assessed the suitability of a repeated dose 28-day oral toxicity study for the identification of metabolic effects of EDCs.

## Materials and methods

### Animals

Male and female wild-type C57BL/6J mice originated from The Jackson Laboratory (BPA and PFOA experiments; RRID:IMSR_JAX:000664) and male and female wild-type C57BL/6N mice originated from the Charles River, Germany (PCN experiments; RRID:MGI:2159965). All procedures involving the animal experiments were approved by the National Animal Experiment Board of Finland (ESAVI/43804/2019, ESAVI/8240/04.10.07/2017, ESAVI/23252/2020) and by the Local Animal Ethics Committee of Wageningen University, the Netherlands (AVD104002015236, 2016.W-0093.024). The studies were performed in accordance with the EU legislation.

### Experimental design

**Fig. 1:**
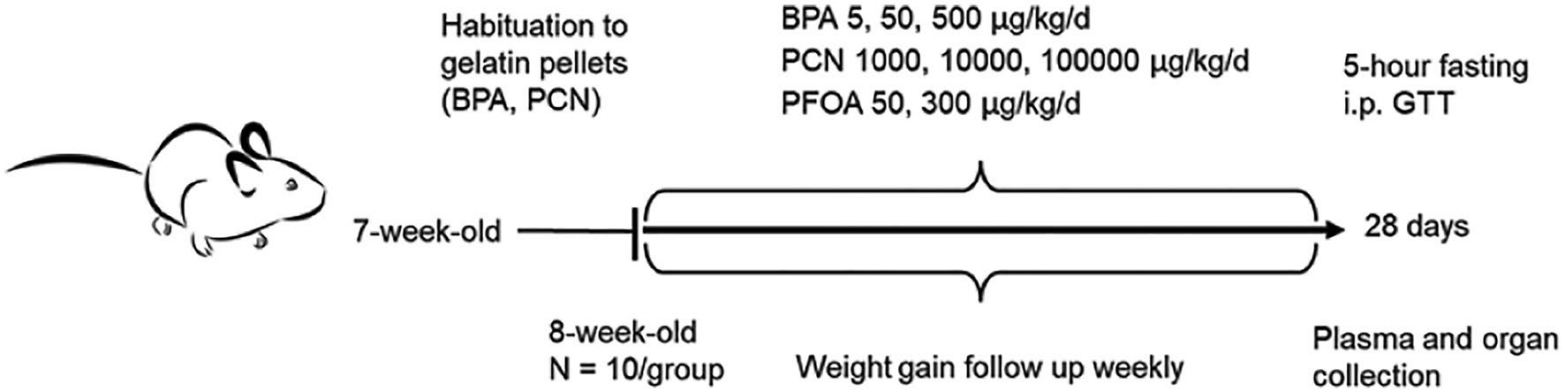
Study timeline

The study was carried out in three locations with three different test substances BPA, PCN, and PFOA. In short, 8–12-week-old mice were randomly divided into experimental groups (*N* = 10) with two (PFOA) or three dose groups (BPA and PCN) next to the control. In the BPA control group, the animal number was 9 due to an unexpected health issue that was not associated with the exposure. At the time of randomization, the average weight of the mice was statistically similar in each group. Male and female mice were included as separate groups in order to account for the potential sexual dimorphism in response to EDCs (McCabe et al. 2017). BPA-exposed animals and their control group were bred and housed in metal cages with plastic-free enrichment and polycarbonate-free drinking bottles to minimize the impact of environmental BPA. The study timeline is presented in Fig. 1.

Animals were exposed to the test compounds for 28 days, in line with OECD’s test guideline 407 (Repeated Dose 28-day Oral Toxicity Study in Rodents) (OECD 2008). The test substances were orally administered either in gelatin pellets (BPA or PCN) (Dhawan et al. 2018) or via drinking water (PFOA). Doses that were applied during the study were 5, 50, and 500 µg/kg body weight/day for BPA, 1000, 10 000, and 100 000 µg/kg body weight/day for PCN, and 50 and 300 µg/kg body weight/day for PFOA. For PFOA two doses were selected based on a previously performed study (Attema et al. 2022). Control groups received gelatin pellets or regular drinking water without the test substances. For mice receiving the gelatin pellets, 1 week of habituation was included prior to the start of the study. During the study period, mice had ad libitum access to regular chow (Inotiv/Envigo; Teklad Global 16% Protein Rodent Diet #TD2016C for BPA; Teklad Global 18% Protein Rodent Diet #TD2018 for PCN; Teklad Global Soy Protein Free-Extruded Rodent Diet #TD2020X for PFOA) and drinking water. #TD2016C and #TD2020X are soy protein free diets while #TD2018 has reduced soy content. The animals were single-housed and maintained under a 12/12-h light/dark cycle. At the end of the 28-day exposure period, intraperitoneal glucose tolerance tests were performed, and the animals were subsequently euthanized according to the standard practices of the local animal facility. For plasma analyses, blood samples were collected from the vena cava from BPA- and PCN-exposed animals, and via orbital puncture from PFOA-exposed animals. Moreover, tissues were weighed and either prepared for histology or snap-frozen in liquid nitrogen prior to storage at –80 °C for subsequent analyses.

### Test compound administration

Mice received gelatin pellets containing PCN (Abcam, #ab144545) or BPA (> 99%, Sigma-Aldrich #239658) every morning and daily administration of the pellet was visually confirmed. Gelatin pellets were prepared by mixing the test compounds with hot blackcurrant juice-gelatin slurry (1.2 g gelatin in 10 ml of 50% juice) and coagulating the mixture in a 96-well microplate. The final volume of the pellet was 160–200 µl, depending on the dose and average body weight of the mice.

For mice receiving PFOA (95%, Sigma-Aldrich #171468), stocks were prepared by dissolving PFOA in drinking water resulting in a final exposure of 50 or 300 µg/kg bw/day. Water intake was measured every week and no differences in water intake between groups were observed. Different stocks were prepared for male and female mice to account for bodyweight differences.

### Intraperitoneal glucose tolerance test

Glucose tolerance was measured via an intraperitoneal glucose tolerance test. For the glucose tolerance test, mice were fasted for 5 h, followed by blood collection via tail bleeding for baseline blood glucose measurements. Subsequently, glucose (1.5 mg/kg body weight) was injected via an intraperitoneal injection after which blood was drawn via tail bleeding after 15, 30, 45, 60, 90, and 120 min for BPA- and PCN-treated mice and 20, 40, 60, 90, and 120 min for PFOA-treated mice. Blood glucose levels were measured by a glucometer.

### Histology

Collected liver tissue was fixed in 4% paraformaldehyde (BPA, PFOA) or 10% neutral buffered formalin (PCN), dehydrated, and embedded in paraffin blocks. Liver sections 5–9 µm thick were stained with Hematoxylin and Eosin (H&E). For Oil-Red-O staining, 5 µM cryosections were air-dried, fixed with formal calcium and stained with Oil-Red-O working solution as described previously (Rakhshandehroo et al. 2007). Sections were subsequently stained with Hematoxylin solution. Images were taken from the section slides with 20 × magnification.

### Plasma lipid measurements

Plasma total cholesterol (CHOL2 cobas c111, Roche; Cholesterol FS assay, DiaSys, Diagnostic Systems GmbH), HDL cholesterol (HDLC4 cobas c111, Roche), LDL cholesterol (LDLC3 cobas c111, Roche) and triglycerides (TRIGL cobas c111, Roche; Liquicolor Mono, Human GmbH) were quantified according to manufacturer’s instructions.

### Liver triglycerides

Liver triglycerides were measured by preparing 5% liver homogenates in a buffer containing sucrose (250 mM), EDTA (2 mM), Tris–base (10 mM) at pH 7.5. Triglycerides were subsequently quantified using a commercially available kit (Liquicolor Mono, Human GmbH) according to manufacturer’s instructions.

### Gene expression analysis

For BPA-treated mice, total RNA was isolated from the liver using NucleoSpin RNA kit (Macherey Nagel) and reversely transcribed with Transcriptor First Strand cDNA Synthesis Kit (Roche). mRNA expression was assayed with PrimeTime Std qPCR Assays (Integrated DNA Technologies) on LightCycler 96 system (Roche). Gene expression data were normalized to *Hprt.*

Total RNA from the livers of PCN-treated mice was isolated using RNAzol RT (Sigma-Aldrich) according to the manufacturer’s instructions. The first strand cDNA was synthesized using random hexamer primers and RevertAid RT kit (Thermo Scientific). Gene expression was analyzed with quantitative real-time PCR using QuantStudio 5 real-time PCR system (Thermo Fisher) and PowerUp SYBR Green Master Mix (Thermo Scientific). Gene expression data were normalized to *Gapdh* and *18S*.

For PFOA-treated mice, RNA was isolated by homogenizing liver tissue using Trizol reagent (Life Technologies), followed by phenol–chloroform-based extraction. cDNA was subsequently synthesized using iScript cDNA synthesis kit (Bio-Rad Laboratories). Gene expression was measured using Sensimix (Bioline) on a CFX384 real-time PCR detection system (Bio-Rad Laboratories). Gene expression data were normalized to *36B4*.

A list of primer sequences used for qPCR analyses can be found in Table 1.

**Table 1:** Sequences of qPCR primers

Gene	Forward primer	Reverse primer
*BPA*		
*mHprt*	5′-CCCCAAAATGGTTAAGGTTGC-3′	5′-AACAAAGTCTGGCCTGTATCC-3′
*mTff3*	5′-TCTGGCTAATGCTGTTGGTG-3′	5′-ACAGTCCACTCTGACATTTGC-3′
*mLifr*	5′-ATCCATAACTTCACCCTGACTG-3′	5′-TCAACGAAGTCGGATCATGAG-3′
*mHmgcr*	5′-ACTGACATGCAGCCGAAG-3′	5′-CACATTCACTCTTGACGCTCT-3′
*mAcc*	5′-AACATCCCCACGCTAAACAG-3′	5′-GTCCAACAGAACATCGCTGA-3′
*mSrebf1*	5′-GTCACTGTCTTGGTTGTTGATG-3′	5′-CGAGATGTGCGAACTGGAC-3′
*mFasn*	5′-GCTCCTCGCTTGTCGTC-3′	5′-ACTCCTGTAGGTTCTCTGACTC-3′
*mNr0b2*	5′-CAAGGCGTATGCGTACCTGAAG-3′	5′-TCCAAGACTTCACACAGTGC-3′
*mCyp2b10*	5′-GAAAGAGGAGTGTGGAGGAG-3′	5′-TGATGCACTGGAAGAGGAAC-3′
*mCyp3a11*	5′-AGTAGCACACTTTCCTTCACC-3′	5′-CCATCTCCATCACAGTATCATACG-3′
*mCyp1a1*	5′-GAACCTTCCCTGATCCTTGTG-3′	5′-TGGAGATTGGGAAAAGCATGA-3′
*mUgt1a1*	5′-CAGAAAAAGCCCCTATCCCA-3′	5′-CCAAAGCCTCAGCAATTTCC-3′
*PCN*		
*18S*	5′-CTCAACACGGGAAACCTCAC-3′	5′-CGCTCCACCAACTAAGAACG-3′
*mGapdh*	5′-GGTCATCATCTCCGCCCC-3′	5′-TTCTCGTGGTTCACACCCATC-3′
*mCyp3a11*	5′-GACAAACAAGCAGGGATGGAC-3′	5′- CCAAGCTGATTGCTAGGAGCA-3′
*mGsta1*	5′-TGTTGAAGAGCCATGGACAA-3′	5′-ATCCATGGGAGGCTTTCTCT-3′
*mPck1*	5′-AGCATTCAACGCCAGGTTC-3′	5′-CGAGTCTGTCAGTTCAATACCAA-3′
*mG6pc*	5′-CGACTCGCTATCTCCAAGTGA-3′	5′-GGGCGTTGTCCAAACAGAAT-3′
*mCd36*	5′-ATGGGCTGTGATCGGAACTG-3′	5′-TTTGCCACGTCATCTGGGTTT-3′
*mPln5*	5′-CTTCCTGCCCATGACTGAG-3′	5′-GACCCCAGACGCACAAAGTA-3′
*mPcsk9*	5′-CCCATCGGGAGATTGAG-3′	5′-TTCCCTTGACAGTTGAGCA-3′
*mSrebf1*	5′-GCAGCCACCATCTAGCCTG-3′	5′-CAGCAGTGAGTCTGCCTTGAT-3′
*mSrebf1a*	5′-CCTGCAGACCCTGGTGAGT-3′	5′-AGAAGACCGGTAGCGCTTCT-3′
*mSrebf1c*	5′-CACAGCCGTGCAGACC-3′	5′-TTGATAGAAGACCGGTAGCGC-3′
*mSrebf2*	5′-TGGGCGATGAGCTGACTCT-3′	5′-CAAATCAGGGAACTCTCCCAC-3′
*mFasn*	5′-GAGGTGGTGATAGCCGGTAT-3′	5′-TGGGTAATCCATAGAGCCCAG-3′
*mAcc*	5′-ATGGGCGGAATGGTCTCTTTC-3′	5′-TGGGGACCTTGTCTTCATCAT-3′
*mScd1*	5′-TTCTTGCGATACACTCTGGTGC-3′	5′-CGGGATTGAATGTTCTTGTCGT-3′
*mAcly*	5′-ACCCTTTCACTGGGGATCACA-3′	5′-GACAGGGATCAGGATTTCCTTG-3′
*mHmgcr*	5′-AGAGCGAGTGCATTAGCAAAG-3′	5′-GATTGCCATTCCACGAGCTA-3′
*mFdps*	5′-GGAGGTCCTAGAGTACAATGCC-3′	5′-AAGCCTGGAGCAGTTCTACAC-3′
*mLdlr*	5′-TCAGACGAACAAGGCTGTC-3′	5′-CATCTAGGCAATCTCGGTCTC-3′
*PFOA*		
*m36b4*	5′-ATGGGTACAAGCGCGTCCTG-3′	5′-GCCTTGACCTTTTCAGTAAG-3′
*mEhhadh*	5′-AAAGCTAGTTTGGACCATACGG-3′	5′-ATGTAAGGCCAGTGGGAGATT-3′
*mCyp4a10*	5′-ACCACAATGTGCATCAAGGAGGCC-3′	5′-AGGAATGAGTGGCTGTGTCGGGGAGAG-3′
*mFatp1*	5′-CGCTTTCTGCGTATCGTCTG-3′	5′-GATGCACGGGATCGTGTCT-3′
*mAcot1*	5′-ATGGCTCTGGCTTATTACA-3′	5′-TAGTTCACGGCTTCTTCA-3′
*Cyp2b10*	5′-AAAGTCCCGTGGCAACTTCC-3′	5′-TTGGCTCAACGACAGCAACT-3′
*Cyp3a11*	5′-CAAGGAGATGTTCCCTGTCA-3′	5′-CCACGTTCACTCCAAATGAT-3′
*mCyp7a1*	5′-GGGATTGCTGTGGTAGTGAGC-3′	5′-GGTATGGAATCAACCCGTTGTC-3′
*mHmgcr*	5′-GTGGCACCGGATGTCTTTG-3′	5′-ACTCTGACCAGATACCACGTT-3′
*mLdlr*	5′-GCATCAGCTTGGACAAGGTGT-3′	5′-GGGAACAGCCACCATTGTTG-3′

### Statistical analysis

Data are presented as mean ± SD. Statistical significance of treatment versus control was determined by one-way ANOVA with Dunnett’s multiple comparison test for normally distributed data or with Kruskal–Wallis test with Dunn’s multiple comparison test for non-normally distributed data. A *P* value < 0.05 was considered statistically significant. Data were visualized and analyzed using GraphPad Prism (GraphPad Software, San Diego, CA, USA). The area under the curve (AUC) from the glucose tolerance tests was calculated using the trapezoidal rule in GraphPad Prism.

## Results

The current study aimed to test the metabolic effects of three different EDCs BPA, PCN, and PFOA, using a standardized 28-day repeated dose oral toxicity test.

**Fig. 2:**
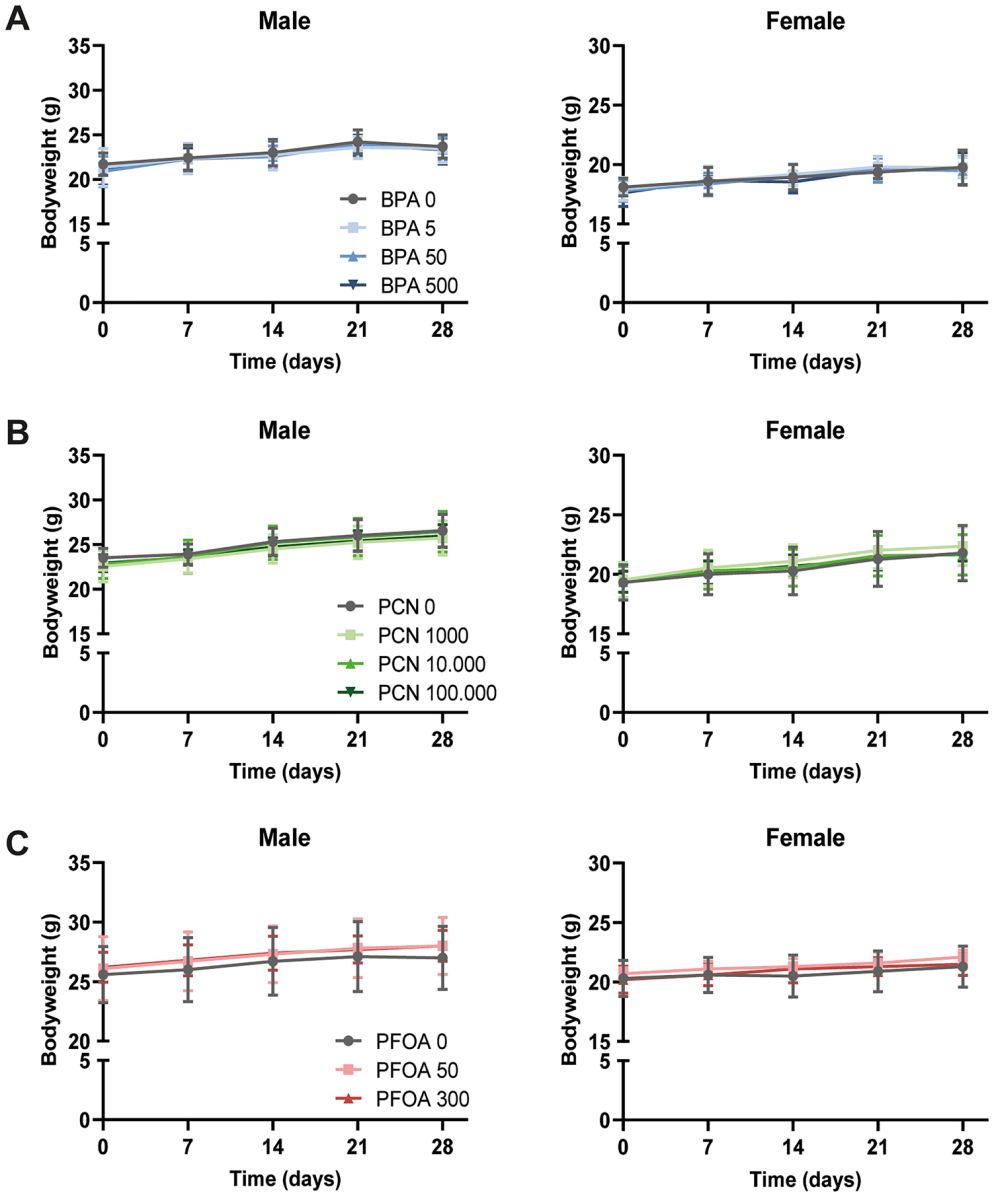
BPA, PCN and PFOA do not affect body weights in male or female mice after 28 days of exposure. Body weight trajectories of male mice and female mice treated with different doses of **A** BPA, **B** PCN and **C** PFOA, concentrations in µg/kg bw/day. *N* = 10, except for BPA 0 female (*N* = 9). Data are depicted as mean ± SD

**Fig. 3:**
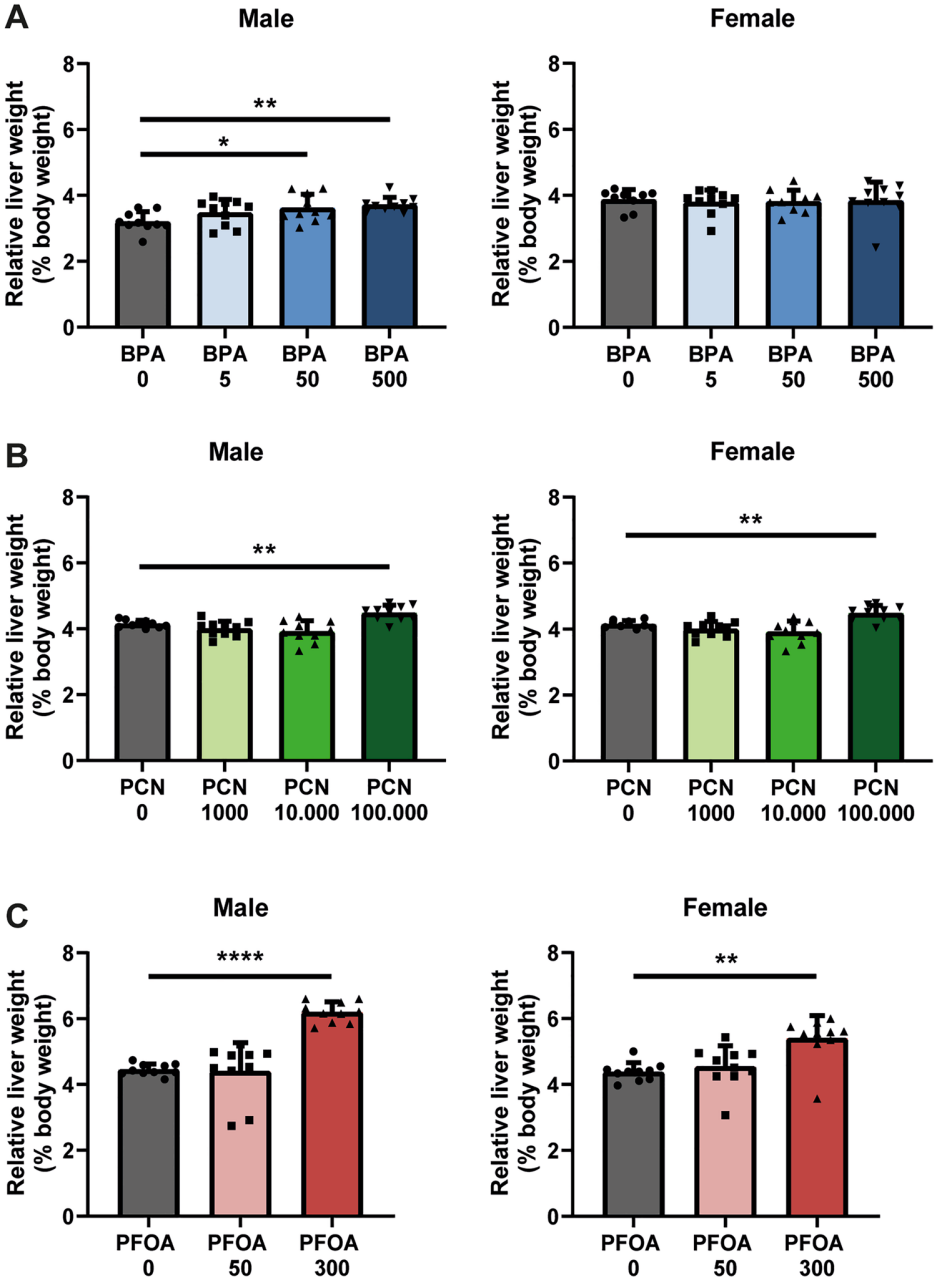
Relative liver weight is increased in mice treated with highest dose of BPA, PCN and PFOA. Liver weights relative to body weight in **A** BPA-treated male and female mice, **B** PCN-treated male and female mice and **C** PFOA-treated male and female mice. Concentrations in µg/kg bw/day. *N* = 10, except for BPA 0 female (*N* = 9). Data are depicted as mean ± SD. Asterisks indicate significant differences between control versus treatment group of male and female mice. *p < 0.05, **p < 0.01, ****p < 0.0001

First, we assessed whether exposure of male and female mice to BPA, PCN, or PFOA for 28 days affected body weights or liver and gonadal adipose tissue (gWAT) weights. After 28 days of exposure, no changes in body weight at either dose were observed in mice exposed to any of the compounds (Fig. 2, Table 2). Exposure of male mice to 50 and 500 µg/kg bw/day BPA significantly increased relative liver weight compared to control mice (Fig. 3a, Table 2). No effects on liver weight were observed in female mice exposed to BPA. In contrast, BPA exposure at all doses decreased gWAT weight in the female but not in the male mice (Table 2). PCN increased relative liver weight in both male and female mice at the highest dose of 100 000 µg/kg bw/day, which, for the female mice, was also reflected in the absolute liver weight (Fig. 3b, Table 2). High-dose PCN did not affect gWAT weights in either sex, however, decrease in relative gWAT weight was observed in males treated with the lowest dose of PCN. Similar to PCN, exposure to PFOA increased absolute and relative liver weight in male and female mice only in the highest dose group (300 µg/kg bw/day) (Fig. 3c, Table 2). Exposure to PFOA did not affect gWAT weight in male or female mice (Table 2).

**Table 2:** Group characteristics and organ weights of BPA-, PCN- and PFOA-treated male and female mice after 28 days of exposure

Experimental group^a^	Mouse strain	Sex	Body weight start (g)	Body weight final (g)	Liver weight (g)	Relative liver weight (% body weight)	gWAT weight (g)	Relative gWAT weight (% body weight)
*BPA*								
	*C57BL6/J*							
BPA 0		M	21.7 ± 1.3	23.7 ± 1.3	0.75 ± 0.08	3.20 ± 0.29	0.25 ± 0.07	1.04 ± 0.40
BPA 5		M	21.3 ± 2.1	23.5 ± 1.6	0.77 ± 0.10	3.48 ± 0.38	0.29 ± 0.10	1.13 ± 0.1
BPA 50		M	20.9 ± 1.7	23.4 ± 1.2	0.82 ± 0.09	3.62 ± 0.40*	0.28 ± 0.06	0.81 ± 0.27
BPA 500		M	21.2 ± 1.8	23.4 ± 1.6	0.82 ± 0.08	3.71 ± 0.22**	0.27 ± 0.09	1.18 ± 0.31
BPA 0		F	18.1 ± 0.8	19.8 ± 1.5	0.76 ± 0.1	3.88 ± 0.28	0.25 ± 0.08	1.29 ± 0.24
BPA 5		F	18.1 ± 1.0	19.7 ± 0.9	0.74 ± 0.08	3.80 ± 0.35	0.18 ± 0.04	0.92 ± 0.41*
BPA 50		F	17.8 ± 0.9	19.5 ± 1.2	0.73 ± 0.1	3.82 ± 0.32	0.18 ± 0.06*	0.91 ± 0.12*
BPA 500		F	17.6 ± 1.1	19.6 ± 1.4	0.73 ± 0.11	3.84 ± 0.53	0.17 ± 0.07*	0.89 ± 0.17*
*PCN*								
	*C57BL6/N*							
PCN 0		M	23.5 ± 1.1	26.6 ± 1.8	1.12 ± 0.12	4.26 ± 0.23	0.48 ± 0.12	1.79 ± 0.32
PCN 1000		M	22.6 ± 1.8	25.7 ± 1.9	1.06 ± 0.16	4.16 ± 0.53	0.35 ± 0.12	1.36 ± 0.37*
PCN 10000		M	22.8 ± 1.6	26.5 ± 2.3	1.15 ± 0.15	4.36 ± 0.30	0.48 ± 0.19	1.80 ± 0.56
PCN 100000		M	22.9 ± 0.9	26.0 ± 1.3	1.23 ± 0.08	4.80 ± 0.28**	0.39 ± 0.11	1.51 ± 0.33
PCN 0		F	19.3 ± 1.5	21.8 ± 2.3	0.84 ± 0.09	4.15 ± 0.11	0.20 ± 0.11	0.94 ± 0.41
PCN 1000		F	19.5 ± 1.5	22.4 ± 1.6	0.84 ± 0.07	4.00 ± 0.23	0.27 ± 0.14	1.26 ± 0.55
PCN 10000		F	19.4 ± 1.3	21.6 ± 1.7	0.80 ± 0.07	3.92 ± 0.32	0.23 ± 0.09	1.09 ± 0.36
PCN 100000		F	19.4 ± 0.9	21.8 ± 0.7	0.92 ± 0.05*	4.48 ± 0.23**	0.22 ± 0.03	1.06 ± 0.13
*PFOA*								
	*C57BL6/J*							
PFOA 0		M	25.6 ± 2.4	27.0 ± 2.7	1.20 ± 0.10	4.45 ± 0.16	0.71 ± 0.42	2.52 ± 1.15
PFOA 50		M	26.1 ± 2.7	28.0 ± 2.4	1.22 ± 0.22	4.40 ± 0.82	0.55 ± 0.14	1.96 ± 0.50
PFOA 300		M	26.2 ± 1.3	28.0 ± 1.3	1.74 ± 0.13****	6.20 ± 0.30****	0.47 ± 0.07	1.68 ± 0.26
PFOA 0		F	20.3 ± 1.5	21.3 ± 1.7	0.93 ± 0.08	4.38 ± 0.27	0.27 ± 0.10	1.23 ± 0.42
PFOA 50		F	20.7 ± 0.5	22.1 ± 0.6	1.00 ± 0.13	4.54 ± 0.60	0.32 ± 0.07	1.43 ± 0.33
PFOA 300		F	20.2 ± 1.1	21.5 ± 0.9	1.07 ± 0.35**	5.04 ± 1.56**	0.24 ± 0.06	1.10 ± 0.29

*p < 0.05, **p < 0.01, ***p < 0.001, ****p < 0.0001

^a^Concentrations in µg/kg bw/day. *N* = 10, except for BPA 0 female (*N* = 9). Data are depicted as mean ± SD. Asterisks indicate significant differences between control versus treatment group of male and female mice

**Fig. 4:**
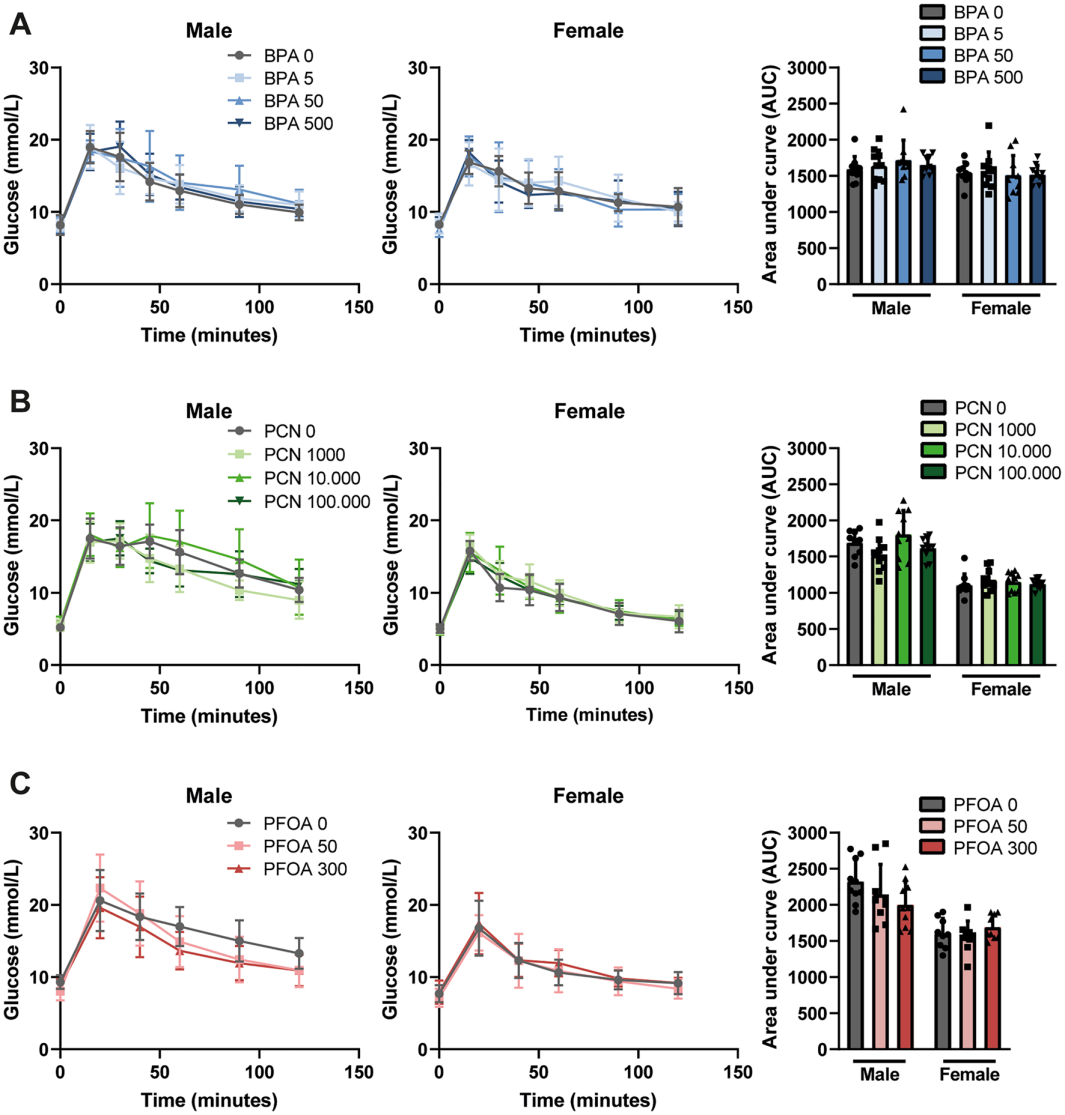
Glucose tolerance is not affected by BPA, PCN and PFOA in male or female mice. Intraperitoneal glucose tolerance tests (1.5 g glucose/kg body weight) after 28 days of treatment and area under the curve (AUC) in **A** BPA-treated male and female mice, **B** PCN-treated male and female mice, and **C** PFOA-treated male and female mice. Concentrations in µg/kg bw/day. *N* = 10, except for BPA 0 female (*N* = 9). Data are depicted as mean ± SD

Next, we tested whether the selected study compounds influence glucose tolerance after 28 days of exposure by performing intraperitoneal glucose tolerance tests (Fig. 4). No effect on glucose tolerance was observed in any of the exposure groups compared to the controls. In addition, none of the study compounds had any effect on fasting glucose (Table 3). Basal fasting glucose levels were higher in the BPA and PFOA experiments (both for the control and test compound-treated mice) compared to the PCN experiment (Table 3). This difference is likely due to the different C57BL/6 substrain that was used in the PCN experiment.

**Table 3:** Plasma metabolites after 28 days of exposure to BPA, PCN and PFOA

Experimental group^a^	Sex	Fasting glucose (mmol/l)	Triglycerides total (mmol/l)	Cholesterol total (mmol/l)	HDL (mmol/l)	LDL (mmol/l)
*BPA*						
BPA 0	M	8.20 ± 1.38	0.71 ± 0.23	1.38 ± 0.29	–	–
BPA 5	M	8.16 ± 0.81	0.74 ± 0.19	1.28 ± 0.22	–	–
BPA 50	M	8.29 ± 1.07	0.72 ± 0.14	1.22 ± 0.20	–	–
BPA 500	M	7.95 ± 1.06	0.77 ± 0.13	1.37 ± 0.19	–	–
BPA 0	F	8.26 ± 0.40	0.74 ± 0.09	1.04 ± 0.21	–	–
BPA 5	F	8.40 ± 1.39	0.71 ± 0.14	1.17 ± 0.26	–	–
BPA 50	F	7.54 ± 1.00	0.71 ± 0.10	1.01 ± 0.23	–	–
BPA 500	F	7.91 ± 1.37	0.72 ± 0.15	1.14 ± 0.17	–	–
*PCN*						
PCN 0	M	5.20 ± 0.58	0.85 ± 0.18	2.11 ± 0.28	1.69 ± 0.19	0.62 ± 0.07
PCN 1000	M	5.50 ± 0.82	0.93 ± 0.18	1.92 ± 0.32	1.45 ± 0.16	0.53 ± 0.08
PCN 10000	M	5.76 ± 0.95	0.80 ± 0.22	2.22 ± 0.35	1.67 ± 0.31	0.61 ± 0.14
PCN 100000	M	5.63 ± 0.68	0.88 ± 0.23	2.20 ± 0.30	1.59 ± 0.25	0.56 ± 0.09
PCN 0	F	5.07 ± 0.60	0.52 ± 0.13	1.32 ± 0.24	1.02 ± 0.20	0.38 ± 0.06
PCN 1000	F	4.77 ± 0.55	0.48 ± 0.10	1.21 ± 0.25	0.99 ± 0.25	0.32 ± 0.05
PCN 10000	F	4.63 ± 0.59	0.54 ± 0.14	1.39 ± 0.33	0.94 ± 0.31	0.34 ± 0.08
PCN 100000	F	4.93 ± 0.62	0.47 ± 0.08	1.43 ± 0.19	0.91 ± 0.10	0.31 ± 0.04*
*PFOA*						
PFOA 0	M	9.31 ± 0.96	1.52 ± 0.31	2.87 ± 0.28	–	–
PFOA 50	M	8.04 ± 1.25	1.06 ± 0.34**	2.37 ± 0.63	–	–
PFOA 300	M	8.74 ± 1.04	1.00 ± 0.16***	2.57 ± 0.40	–	–
PFOA 0	F	7.72 ± 1.19	0.96 ± 0.24	2.12 ± 0.21	–	–
PFOA 50	F	7.14 ± 1.24	0.88 ± 0.20	1.92 ± 0.25	–	–
PFOA 300	F	7.90 ± 1.60	0.64 ± 0.07**	1.81 ± 0.31*	–	–

*p < 0.05, **p < 0.01, ***p < 0.001, ****p < 0.0001

^a^Levels of plasma triglycerides, total cholesterol, HDL, LDL and fasting blood glucose levels after 28 exposure to BPA, PCN and PFOA in male and female mice. Concentrations in µg/kg bw/day. *N* = 10, except for BPA 0 female (*N* = 9). Data are depicted as mean ± SD. Asterisks indicate significant differences between control vs treatment group of male and female mice

**Fig. 5:**
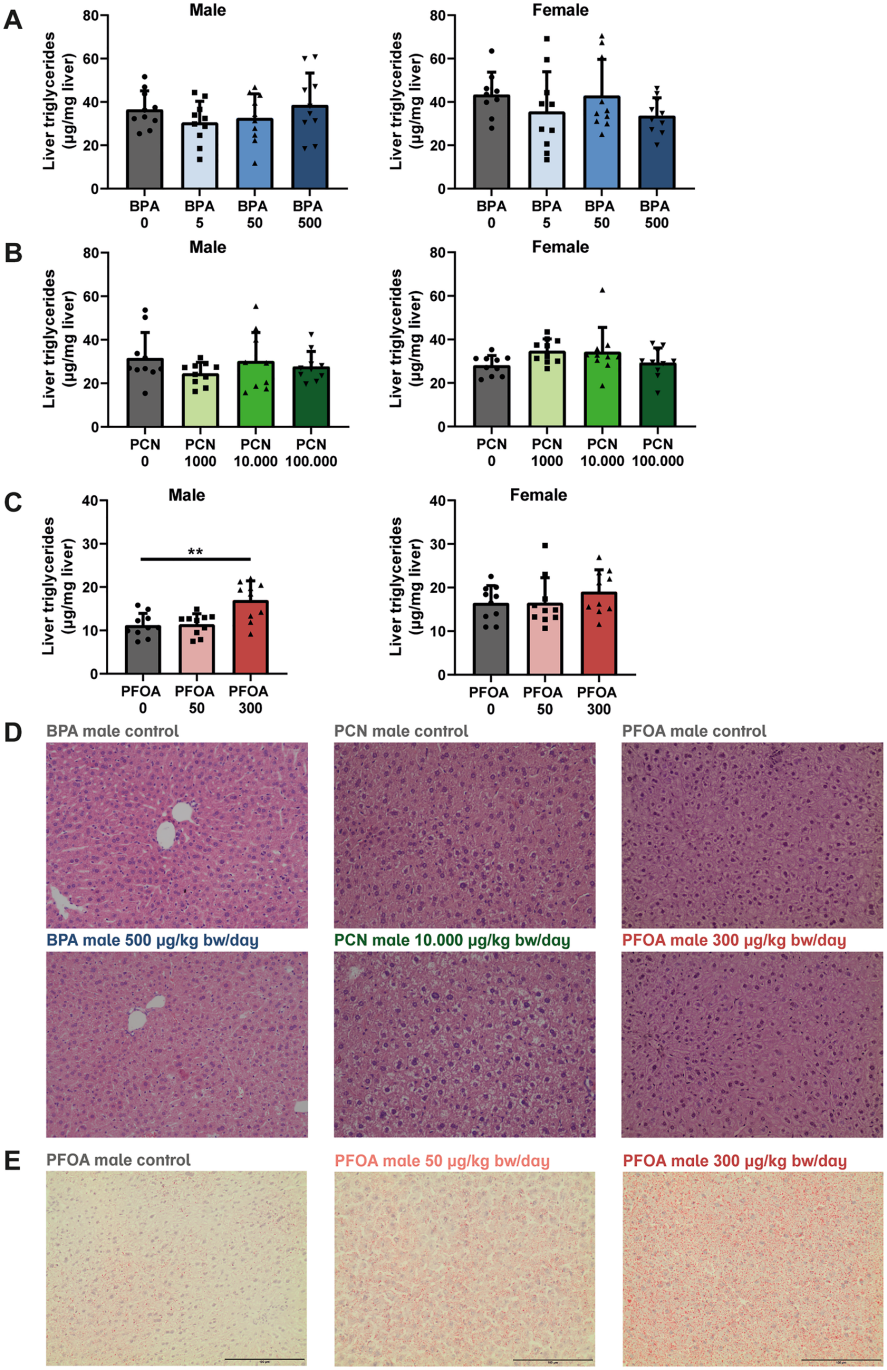
Hepatic triglycerides in BPA-, PCN- and PFOA-treated male and female mice. Measurement of triglycerides in liver of **A** BPA-treated male and female mice, **B** PCN-treated male and female mice and **C** PFOA-treated male and female mice. **D** H&E staining of representative liver Sects. (20 × magnification) of male control mice and mice treated with highest dose of BPA, PCN or PFOA. **E** Oil-Red-O staining from PFOA-treated male mice. Concentrations in µg/kg bw/day. *N* = 10, except for BPA 0 female (*N* = 9). Data is depicted as mean ± SD. Asterisks indicate significant differences between control versus treatment group of male and female mice. **p < 0.01

Many EDCs with established metabolism-disrupting capacities are known to affect plasma and hepatic lipids (Heindel et al. 2017). Therefore, we tested the ability of BPA, PCN, and PFOA to alter plasma triglycerides, plasma total cholesterol, and hepatic triglyceride levels after 28 days of exposure. Because recent reports indicate regulation of cholesterol and lipoprotein metabolism by PXR (Gwag et al. 2019; Karpale et al. 2021), plasma HDL- and LDL cholesterol were additionally measured for PCN-treated mice. Exposure to up to 500 µg/kg bw/day BPA or 100 000 µg/kg bw/day PCN did not result in changes in plasma triglycerides or total cholesterol in either male or female mice (Table 3). Plasma HDL cholesterol was not affected by PCN. However, plasma LDL cholesterol was lower in female mice exposed to the highest dose of PCN compared to the controls, but this effect could not be detected in male mice (Table 3). Furthermore, hepatic triglyceride levels were not affected by BPA or PCN (Fig. 5a, b). Exposure to PFOA significantly decreased plasma triglycerides at either dose for male mice. For female mice, the reduction in plasma triglycerides was only observed in the high-dose group. Of interest, total plasma cholesterol levels were significantly decreased with the high dose of PFOA (300 µg/kg bw/day) in female but not in male mice. Next to that, PFOA at 300 µg/kg bw/day significantly increased hepatic triglycerides in the male mice, which was not observed in the lower dose group or the female mice (Fig. 5c).

H&E staining of liver sections from the BPA-, PCN-, or PFOA-treated male mice did not reveal obvious differences in hepatic triglyceride content (Fig. 5d). As the quantitative analysis indicated an increase in triglyceride content in the livers of male mice exposed to PFOA (Fig. 5c), Oil red O staining was performed on these sections. Oil red O staining confirmed the quantitative triglyceride measurement, showing increased lipid accumulation in male mice exposed to PFOA (Fig. 5e). Overall, BPA and PCN did not significantly influence plasma triglycerides, cholesterol, or hepatic triglycerides after 28 days of treatment, except for the minor effect of the highest PCN dose on plasma LDL cholesterol. However, exposure to high-dose PFOA reduced plasma triglycerides and increased triglyceride content in the liver.

Endocrine-disrupting effects of BPA, PCN, and PFOA are thought to be mediated predominantly via ERs, PXR, and PPARα, respectively. Accordingly, we measured the gene expression levels of some established target genes in the liver to test whether these nuclear receptors were activated by the treatments (Tables 4, 5, 6). First, the expression of estrogen-sensitive genes *Tff3* and *Lifr* was assessed in the BPA-exposed animals. Overall, there was a tendency for BPA to induce *Tff3*, but the effect was statistically significant only for male mice exposed to 500 µg/kg bw/day BPA (Table 4). Activation of PXR by PCN was confirmed by induction of the classical target genes *Cyp3a11* and *Gsta1* (Table 5). However, only exposure to the highest dose of PCN resulted in a significant induction of *Cyp3a11* and *Gsta1* in both male and female mice. In addition, *Cyp3a11* was induced in males also with the second highest dose of PCN (Table 5). Exposure to PFOA resulted in clear activation of PPARα reflected by a pronounced increase in expression of the well-known PPARα target genes *Ehhadh*, *Acot1*, *Fatp1,* and *Cyp4a10* in livers of both male and female mice receiving the high dose of PFOA (Table 6). Next to that, we observed an increase in the expression of *Cyp2b10* and *Cyp3a11* in these mice, suggesting CAR and PXR activation following exposure to PFOA. The upregulation of *Cyp3a11* was also evident in the lower dose group of 50 µg/kg bw/day PFOA.

**Table 4:** Effect of BPA on hepatic expression of ER-sensitive genes and selected metabolic genes in male and female mice

	Male					Female			
	BPA 0	BPA 5	BPA 50	BPA 500		BPA 0	BPA 5	BPA 50	BPA 500
Estrogen-sensitive genes									
*Tff3*	1.00 ± 0.62	2.02 ± 1.76	1.63 ± 0.78	2.42 ± 1.97*		1.00 ± 0.69	2.74 ± 3.50	4.04 ± 5.01	3.39 ± 5.68
*Lifr*	1.00 ± 0.37	1.35 ± 0.27	1.17 ± 0.37	1.27 ± 0.29		1.00 ± 0.28	0.9 ± 0.22	0.94 ± 0.54	0.96 ± 0.20
Cholesterol metabolism									
*Hmgcr*	1.00 ± 0.47	1.66 ± 0.73	1.03 ± 0.84	1.10 ± 0.55		1.00 ± 0.29	1.92 ± 0.78**	0.60 ± 0.30	1.12 ± 0.28
Lipid metabolism									
*Acc*	1.00 ± 0.34	1.38 ± 0.68	0.91 ± 0.27	0.91 ± 0.22		1.00 ± 0.38	0.8 ± 0.19	0.58 ± 0.19**	0.74 ± 0.25
*Srebf1*	1.00 ± 0.29	1.47 ± 0.33*	1.38 ± 1.04	1.00 ± 0.29		1.00 ± 0.12	1.09 ± 0.29	0.66 ± 0.22	0.92 ± 0.22
*Fasn*	1.00 ± 0.38	1.73 ± 1.10	1.10 ± 0.55	1.06 ± 0.38		1.00 ± 0.38	1.09 ± 0.47	0.52 ± 0.21**	0.82 ± 0.44
*Nr0b2*	1.00 ± 0.38	1.17 ± 0.42	1.09 ± 0.59	1.19 ± 0.42		1.00 ± 0.22	1.33 ± 0.46	1.05 ± 0.56	1.18 ± 0.58
CAR and PXR target genes									
*Cyp2b10*	1.00 ± 0.69	0.92 ± 0.58	0.85 ± 0.80	0.71 ± 0.55		1.00 ± 0.49	0.94 ± 0.38	0.54 ± 0.20*	0.79 ± 0.38
*Cyp3a11*	1.00 ± 0.17	0.92 ± 0.22	0.91 ± 0.25	0.87 ± 0.21		1.00 ± 0.17	0.94 ± 0.14	0.89 ± 0.32	0.86 ± 0.09*
Xenobiotic metabolism									
*Cyp1a1*	1.00 ± 0.25	1.25 ± 0.36	0.85 ± 0.25	1.03 ± 0.49		1.00 ± 0.65	0.49 ± 0.11*	0.60 ± 0.19	0.67 ± 0.13
*Ugt1a1*	1.00 ± 0.16	1.00 ± 0.1	0.95 ± 0.15	0.86 ± 0.16		1.00 ± 0.10	0.86 ± 0.15	0.78 ± 0.25*	0.92 ± 0.15

Hepatic gene expression of BPA-treated male and female mice after 28 days of exposure. Data are normalized to *Hprt* as housekeeping gene and expressed relative to male or female control. Concentrations in µg/kg bw/day. *N* = 10, except for BPA 0 female (*N* = 9). Data are depicted as mean ± SD. Asterisks indicate significant differences between control vs treatment group of male and female mice

*p < 0.05, **p < 0.01

**Table 5:** Effect of PCN on hepatic expression of PXR target genes and genes involved in lipid and cholesterol metabolism in male and female mice

	Male				Female			
	PCN	PCN	PCN	PCN	PCN	PCN	PCN	PCN
	0	1000	10 000	100 000	0	1000	10 000	100 000
PXR target genes								
*Cyp3a11*	1.00 ± 0.22	1.09 ± 0.23	1.69 ± 0.83*	5.92 ± 3.06****	1.00 ± 0.26	1.17 ± 0.23	1.23 ± 0.23	3.90 ± 1.86****
*Gsta1*	1.00 ± 0.50	1.08 ± 0.57	1.21 ± 0.59	5.01 ± 4.56****	1.00 ± 0.56	1.14 ± 0.91	2.76 ± 2.75	9.54 ± 8.12****
Glucose metabolism								
*Pck1*	1.00 ± 0.28	1.25 ± 0.25	1.46 ± 0.29**	1.46 ± 0.35**	1.00 ± 0.33	0.95 ± 0.21	1.05 ± 0.23	0.93 ± 0.22
*G6pc*	1.00 ± 0.54	0.95 ± 0.26	1.43 ± 0.68	1.25 ± 0.5	1.00 ± 0.44	0.78 ± 0.26	0.67 ± 0.20*	0.56 ± 0.23**
Lipid and cholesterol metabolism								
*Cd36*	1.00 ± 0.51	0.72 ± 0.37	1.23 ± 0.95	0.90 ± 0.80	1.00 ± 0.50	0.56 ± 0.37*	0.58 ± 0.36	0.57 ± 0.25
*Pln5*	1.00 ± 0.25	0.91 ± 0.20	1.06 ± 0.20	0.78 ± 0.16	1.00 ± 0.27	0.84 ± 0.19	1.00 ± 0.33	0.69 ± 0.21*
*Pcsk9*	1.00 ± 0.51	2.34 ± 0.97*	2.29 ± 1.26	2.56 ± 1.55*	1.00 ± 0.57	1.43 ± 0.60	2.05 ± 1.59	1.70 ± 0.99
*Srebf1*	1.00 ± 0.41	1.11 ± 0.27	1.20 ± 0.32	1.02 ± 0.43	1.00 ± 0.34	1.15 ± 0.50	0.99 ± 0.39	1.18 ± 0.57
*Srebf1a*	1.00 ± 0.39	0.97 ± 0.25	1.07 ± 0.40	0.90 ± 0.39	1.00 ± 0.58	0.88 ± 0.29	0.77 ± 0.29	0.89 ± 0.36
*Srebf1c*	1.00 ± 0.34	1.02 ± 0.29	1.02 ± 0.30	0.87 ± 0.42	1.00 ± 0.38	1.02 ± 0.32	0.87 ± 0.34	1.04 ± 0.47
*Srebf2*	1.00 ± 0.18	1.14 ± 0.25	0.97 ± 0.26	0.97 ± 0.27	1.00 ± 0.18	1.02 ± 0.23	1.08 ± 0.19	1.02 ± 0.24
*Fasn*	1.00 ± 0.55	0.97 ± 0.37	1.15 ± 0.38	0.97 ± 0.50	1.00 ± 0.25	0.95 ± 0.26	0.97 ± 0.22	1.20 ± 0.37
*Acc*	1.00 ± 0.27	1.20 ± 0.35	1.26 ± 0.21	0.99 ± 0.16	1.00 ± 0.20	0.95 ± 0.25	0.99 ± 0.22	0.97 ± 0.26
*Scd1*	1.00 ± 0.42	0.99 ± 0.39	1.26 ± 0.78	0.81 ± 0.29	1.00 ± 0.37	0.87 ± 0.33	1.34 ± 0.71	1.15 ± 0.48
*Acly*	1.00 ± 0.45	1.40 ± 1.07	1.38 ± 0.61	1.27 ± 1.52	1.00 ± 0.17	1.09 ± 0.49	0.93 ± 0.23	1.10 ± 0.37
*Hmgcr*	1.00 ± 0.31	1.27 ± 0.49	1.16 ± 0.65	1.14 ± 0.95	1.00 ± 0.24	1.03 ± 0.52	1.31 ± 0.61	1.26 ± 0.55
*Fdps*	1.00 ± 0.40	1.32 ± 0.57	1.21 ± 0.48	1.20 ± 0.57	1.00 ± 0.21	0.98 ± 0.33	1.09 ± 0.39	1.20 ± 0.48
*Ldlr*	1.00 ± 0.19	1.17 ± 0.38	1.13 ± 0.39	1.14 ± 0.40	1.00 ± 0.17	1.06 ± 0.26	1.14 ± 0.25	1.10 ± 0.26

Hepatic gene expression of PCN-treated male and female mice after 28 days of exposure. Data are normalized to *Gapdh* and *18s* as housekeeping genes and expressed relative to male or female control. Concentrations in µg/kg bw/day. *N* = 10. Data are depicted as mean ± SD. Asterisks indicate significant differences between control vs treatment group of male and female mice

*p < 0.05, **p < 0.01, ***p < 0.001, ****p < 0.0001

**Table 6:** Effect of PFOA on hepatic expression of PPARα target genes, CAR/PXR target genes and selected genes involved cholesterol metabolism in male and female mice

	Male			Female		
	PFOA	PFOA	PFOA	PFOA	PFOA	PFOA
	0	50	300	0	50	300
PPARα target genes/lipid metabolism						
*Ehhadh*	1.00 ± 0.37	1.96 ± 0.42	15.96 ± 3.99****	1.00 ± 0.39	1.38 ± 0.34	9.28 ± 3.86****
*Cyp4a10*	1.00 ± 0.74	5.03 ± 2.94	24.84 ± 7.91****	1.00 ± 0.54	2.90 ± 1.21*	18.97 ± 5.37****
*Acot1*	1.00 ± 0.35	2.87 ± 0.80****	17.42 ± 6.66****	1.00 ± 0.72	1.61 ± 0.59	13.49 ± 4.56****
*Fatp1*	1.00 ± 0.40	1.03 ± 0.44	7.66 ± 2.53****	1.00 ± 0.25	1.24 ± 0.39	5.12 ± 2.00****
CAR and PXR target genes						
*Cyp2b10*	1.00 ± 0.82	1.16 ± 0.53	2.51 ± 1.28**	1.00 ± 0.61	1.42 ± 0.64	2.61 ± 0.75****
*Cyp3a11*	1.00 ± 0.29	2.45 ± 0.74****	3.09 ± 0.53****	1.00 ± 0.28	1.93 ± 0.45****	2.47 ± 0.41****
Cholesterol metabolism						
*Cyp7a1*	1.00 ± 0.64	0.68 ± 0.34	0.36 ± 0.21***	1.00 ± 0.79	1.86 ± 1.00*	0.55 ± 0.28
*Hmgcr*	1.00 ± 0.32	0.89 ± 0.38	1.45 ± 0.40*	1.00 ± 0.19	1.18 ± 0.39	0.98 ± 0.30
*Ldlr*	1.00 ± 0.43	1.03 ± 0.32	1.12 ± 0.28	1.00 ± 0.33	1.25 ± 0.34	1.01 ± 0.32

Hepatic gene expression of PFOA-treated male and female mice after 28 days of exposure. Data are normalized to *36b4* as housekeeping gene and expressed relative to male or female control. Concentrations in µg/kg bw/day. *N* = 10. Data are depicted as mean ± SD. Asterisks indicate significant differences between control vs treatment group of male and female mice

*p < 0.05, **p < 0.01, ***p < 0.001, ****p < 0.0001

We also measured liver mRNA expression of selected genes involved in lipid, glucose, or xenobiotic metabolism. In the male mice, we observed an increase in the expression of *Srebf1* in the 5 µg/kg bw/day BPA dose group. In the female mice, the expression levels of *Fasn, Acc*, *Cyp2b10,* and *Ugt1a1* were decreased in the 50 µg/kg bw/day dose group, while *Hmgcr* and *Cyp1a1* were up- and downregulated, respectively, by the lowest dose of BPA. Moreover, we also observed a slight but statistically significant decrease in the expression of *Cyp3a11* by the highest dose of BPA if the female mice (Table 4).

The highest dose of PCN altered the expression of genes involved in glucose metabolism (*Pck1* induced in males, *G6pc* repressed in females), lipid metabolism, and cholesterol biosynthesis (*Cd36* and *Pln5* repressed in females, *Pcsk9* induced in males). Some of these effects were detected also with the lower doses. Several other genes previously reported to be PXR responsive in certain conditions and related to lipogenesis and cholesterol metabolism were also analyzed, including S*rebf1*, *Srebf1a*, *Srebf1c*, *Screbf2*, *Fasn*, *Acc*, *Scd1*, *Acly*, *Hmgcr*, *Fdps,* and *Ldlr,* but were not affected by PCN treatment (Table 5).

Since PFOA has been linked to alterations in cholesterol metabolism (Schlezinger et al. 2020; Liu et al. 2023), the expression of genes involved in different branches of cholesterol metabolism was assessed. Of interest, a downregulation in the expression of *Cyp7a1*, which is involved in the conversion of cholesterol into bile acids, was observed in male mice exposed to the highest dose of PFOA. At the same time, exposure to PFOA in these mice resulted in a small but significant induction of *Hmgcr*. The expression of *Ldlr* was not affected by PFOA at the indicated doses in either male or female mice (Table 6).

## Discussion

Identification of MDCs and understanding their mode of action is important for the prevention of human metabolic diseases. Currently, there are limited tools available for the identification of MDCs in toxicity testing to support risk assessment. In the current study, we assessed the metabolic effects of three compounds with suggested metabolism-disrupting capacities according to the guidelines for a repeated dose 28-day oral exposure study (OECD 2008). We did not observe strong effects of the three compounds on overall body weight, glucose tolerance, plasma lipid levels, and hepatic triglyceride levels.

In the current study, limited metabolic effects were detected in BPA-exposed animals. Primary phenotypic changes observed include an increased liver weight in male mice and decreased gWAT weight in female mice. Further, the observed increase in the liver weights of the male mice was not accompanied by increased triglyceride content, nor by alterations in glucose tolerance. Moreover, the hepatic gene expression changes were mostly seen with non-monotonic dose responses. The low-dose effects and U-shaped dose–response relationships have been previously shown for several BPA-induced effects (Jenkins et al. 2011; Marmugi et al. 2012; Angle et al. 2013; Villar-Pazos et al. 2017). In addition, differential gene expression during various time points of BPA exposure in adult rodents has been described (Ke et al. 2016). For example, epigenetic modifications have been suggested to play a role in these effects (Brulport et al. 2020). However, the exact mechanisms for the suggested low-dose effects remain elusive. Based on the existing data and the lack of prominent effects in this study, it is reasonable to conclude that BPA does not cause prominent metabolic effects in C57BL/6J mice after 28 days of repeated low-dose oral exposure. It is possible that certain mouse strains such as CD-1 are more susceptible to BPA effects in the liver compared to C57BL/6J (Marmugi et al. 2012).

With regards to PCN, the metabolic effects in mice following exposure were modest compared to previous findings with the same compound but in a different experimental setting (Rysä et al. 2013; Spruiell et al. 2014; Ling et al. 2016; Hassani-Nezhad-Gashti et al. 2018). For example, we have shown previously that 4-day intraperitoneal treatment with PCN (50 mg/kg bw/day) impairs glucose tolerance (Hassani-Nezhad-Gashti et al. 2018). Importantly, a similar effect was observed after 1 week of rifampicin (human PXR ligand) treatment in humans (Rysä et al. 2013). We now used 28-day oral PCN exposure to mimic a more physiological route of exposure. PXR target genes *Cyp3a11* and *Gsta1* were significantly induced by the highest treatment dose indicating successful exposure to PCN and PXR activation in the mouse liver. However, glucose tolerance was not impaired. In our previous study, glucose was administered by oral gavage (Hassani-Nezhad-Gashti et al. 2018). Instead, in this study, we used intraperitoneal glucose administration as a standardized protocol for the three compounds studied in the different laboratories. The different administration routes of PCN and glucose may have contributed to the discrepant results in glucose tolerance tests. Oral glucose administration has been shown to be more physiological, while *i.p.* injection resulted in higher blood glucose and a lack of a peak in insulin secretion (Small et al. 2022). We observed significant changes in gluconeogenic genes *Pck1* and *G6pc* after PCN exposure. *Pck1* was induced in male mice and *G6pc* was repressed in female mice exposed to the two highest doses of PCN. The metabolic consequences of these changes in gene expression are unclear.

There were no differences in body weight, but we observed an increased liver-to-body weight ratio in both genders after the highest PCN dose, and increased liver weight in the male mice. PXR activation is known to cause liver enlargement (Jiang et al. 2019). Similar to BPA treatment, the increased liver weight in PCN-treated mice was not associated with increased liver triglyceride content. However, several previous studies have observed PXR-induced liver steatosis (Gwag et al. 2019; Nakamura et al. 2007; Zhou et al. 2006, 2008). In addition, several lipogenic genes have been described to be induced by PXR ligands (Nakamura et al. 2007; Zhou et al. 2006, 2008), which was not detected in the current study. Only very limited changes in plasma lipids were observed after PCN treatment. In the females exposed to the highest PCN dose plasma LDL cholesterol was slightly lower compared to the controls. This contrasts with previous studies describing a hypercholesterolemic effect of PXR activation in mice and humans (Gwag et al. 2019; Karpale et al. 2021). We have previously observed a widespread induction of genes of the cholesterol synthesis pathway as well as *PCSK9* in obese mice treated 4 days i.p. with PCN (Karpale et al. 2021). In the current experimental setup, only *Pcsk9* expression was slightly induced by PCN. Overall, the PCN effect on metabolic health parameters was very minor in the current study setting. This was unexpected considering the ample evidence of metabolism-disrupting effects both in rodent models and in human clinical studies (Hukkanen and Hakkola 2020). A limitation to the PCN experiment was that the chow diet was not totally soy free but reduced soybean meal with moderate levels of isoflavones that many have some endocrine activity. Nevertheless, this exposure was similar in the control and PCN-treated groups.

Exposure to PFOA for 28 days resulted in an induction of liver weight and a reduction in plasma triglycerides in both male and female mice. Next to that, male mice receiving PFOA showed increased hepatic triglyceride content. In line with this, a pronounced upregulation of PPARα target genes was observed in male and female mouse livers. The activation of PPARα following PFOA exposure is consistent with previous observations in various in vivo and in vitro models (DeWitt et al. 2008; Wolf et al. 2008; Rosen et al. 2017; Behr et al. 2020b; Schlezinger et al. 2021; Attema et al. 2022). In addition, the increase in hepatic triglycerides in response to PFOA has been shown in both mice as well as in human hepatocytes (Attema et al. 2022; Louisse et al. 2020; Schlezinger et al. 2021). We previously found the gene expression changes induced by PFOA in mouse liver to be 88% dependent on PPARα indicating a large role of PPARα in mediating the metabolic effects by PFOA in liver (Attema et al. 2022). Indeed, the hypolipidemic effects observed by PFOA mimic the response seen by typical PPARα activators (Staels et al. 1998; Wolf et al. 2008). At the same time, we also observed an induction in hepatic expression of the CAR and PXR target genes *Cyp2b10* and *Cyp3a11*. For *Cyp3a11*, a modest but significant increase was also observed for the lower dose of 50 µg/kg bw/day of PFOA, suggesting that *Cyp3a11* is a sensitive target in response to PFOA exposure. The activation of PXR and CAR by PFOA has been established before (Bjork et al. 2011; Rosen et al. 2017; Attema et al. 2022), and is believed to contribute to the increased hepatic triglyceride content and reduction in plasma triglycerides.

Exposure to high-dose PFOA for 28 days resulted in a reduction of total plasma cholesterol levels in female mice but not in male mice. Previous rodent studies have also observed a reduction in plasma cholesterol in response to PFOA exposure (Wang et al. 2013; Attema et al. 2022). Of interest, we did observe an upregulation of *Hmgcr* and downregulation of *Cyp7a1* in the livers of male mice, which would be expected to result in an increase in plasma cholesterol levels. However, the relationship between PFOA and cholesterol metabolism seems to be intricate, showing different responses in both different types of rodent models as well as in human-based models (Rebholz et al. 2016; Behr et al. 2020a; Louisse et al. 2020; Schlezinger et al. 2020).

Glucose tolerance was not found to be affected in mice after 28 days of exposure to PFOA. This is different from observations by Yan et al. which showed an improvement in glucose tolerance and insulin sensitivity in male Balb/c mice treated with 50 µg/kg bw/day PFOA for 28 days next to a normal chow diet (Yan et al. 2015). However, mice also displayed a reduction in body weight and fat mass. In the current study, no changes in body weight were observed. Considering the well-established interaction between body weight and insulin sensitivity (Kahn and Flier 2000), the effect of PFOA on glucose tolerance is expected to be more indirectly related to the changes in body weight rather than a direct effect of PFOA on glucose metabolism. A similar weight-reducing effect was observed in our previous study combining high-fat diet-fed mice and PFOA treatment (Attema et al. 2022).

The response to EDCs can be sexually dimorphic (McCabe et al. 2017). Indeed, hepatic nuclear receptor activation is known to be differentially regulated between the sexes (Rando and Wahli 2011). We observed slight differences in response to the tested EDCs between male and female mice. For example, male and female mice displayed a different response to BPA in liver or adipose tissue weight gain. In addition, both BPA and PCN exposure resulted in differential gene regulation between male and female mice. For PFOA, the differences between sexes were limited, although the metabolic effects of PFOA tended to be stronger in male mice. Apart from sex-related differences, the animal strain is also known to influence the specific response to EDCs (Marmugi et al. 2012; Rebholz et al. 2016; Schlezinger et al. 2020). In the current study, we observed higher fasting blood glucose levels in C57BL/6J mice than in C57BL/6N mice, which may be caused by genetic differences. The C57BL/6J strain carries a mutation in the mitochondrial *Nnt* gene making this substrain more susceptible to disturbances in glucose metabolism (Toye et al. 2005). Overall, both sex and animal strain can influence the metabolic response to EDCs. For this reason, the selection of the specific animal model to be used in the context of EDCs requires careful consideration.

The study compounds were administered through oral route with two different methods. PFOA was administered in drinking water while for BPA and PCN, because of limited water solubility, we utilized daily voluntary administration with palatable gelatin pellets. Gelatin pellets have been proven to be an effective method for delivering substances via oral route, without causing administration-related stress (Cox et al. 2010; Dhawan et al. 2018; Zhang 2021; Martins et al. 2022). Compared to forced feeding by oral gavage, voluntary administration of the compounds provides better physiological relevance mimicking human dietary exposure (Vandenberg et al. 2014). Alternative to the pellets the drug administration could have been performed also by mixing into chow. Different from the chow-mixture the pellet administration represents a single daily bolus, which results in different toxicokinetics and potentially may also cause some differences in the toxic response. The pellet administration requires single housing of the mice and visual confirmation of administration. Especially C57BL/6J mice have been described to be eager and fast to consume gelatin pellets (Martins et al. 2022). Here, this was further extended to C57BL/6N substrain. However, this method is not suitable for administrating compounds with strong unpleasant taste. A limitation to our study is that plasma concentrations of the study compounds were not measured. Thus, in the case of pellet administration the proof of exposure is based on visual verification of the mice eating the pellet and the expected changes in the gene expression.

Altogether, of the three studied compounds, PFOA induced the most pronounced effects on metabolic endpoints in the current study. However, the effects of all compounds were relatively modest. The limited metabolic effects might be explained by the use of young, low-fat-fed mice. The metabolically healthy mice may be quite resistant to metabolic disruption. High-fat or Western-style diets are routinely used in rodents to induce obesity and related metabolic complications such as insulin resistance, type 2 diabetes, and hepatic steatosis (Cai et al. 2005; Patsouris et al. 2006) and are an excellent model to study disruption of metabolic processes. Thus, a high-fat diet model could be more suitable to potentiate metabolic effects induced by EDCs as shown in previous studies using high-fat diets combined with EDC exposure including PFOA (Schlezinger et al. 2020; Attema et al. 2022), PCN (Karpale et al. 2021) and BPA (Moon et al. 2015).

In conclusion, the current study shows that exposure to BPA-, PFOA-, and PCN-induced limited metabolic effects after 28 days of exposure in male and female C57BL/6 mice. Thus, the repeated dose 28-day oral toxicity protocol may not be a very sensitive approach for studying the metabolic effects of EDCs. Future models should focus on the incorporation of predisposing factors in the experimental setup to increase sensitivity, such as feeding a high-fat diet in conjunction with EDCs.

## Data Availability

The data is available upon request from the corresponding author.

## Abbreviations

AUC: Area under the curve
BPA: Bisphenol A
EDCs: Endocrine-disrupting chemicals
ERα: Estrogen receptor α
ERβ: Estrogen receptor β
ERRγ: Estrogen-related receptor γ
LXR: Liver X receptor
MDCs: Metabolism-disrupting chemicals
NAFLD: Non-alcoholic fatty liver disease
PCN: Pregnenolone-16α-carbonitrile
PFAS: Poly- and perfluoroalkyl substances
PPAR: Peroxisome proliferator-activated receptor
PPARα: Peroxisome proliferator-activated receptor α
PXR: Pregnane X receptor
PFOA: Perfluorooctanoic acid
THR: Thyroid hormone receptor
